# Microbial Community and Its Association With Physicochemical Factors During Compost Bedding for Dairy Cows

**DOI:** 10.3389/fmicb.2020.00254

**Published:** 2020-02-21

**Authors:** Likun Sun, Xiangmin Han, Jianshu Li, Zhidong Zhao, Yuzhen Liu, Qiming Xi, Xinyu Guo, Shuangbao Gun

**Affiliations:** ^1^College of Animal Science, Gansu Agricultural University, Lanzhou, China; ^2^Gansu Provincial Engineering Research Center for Animal Waste Utilization, Gansu Agricultural University, Lanzhou, China; ^3^Grassland Science College of Gansu Agricultural University, Lanzhou, China

**Keywords:** cow manure, compost bedding, high throughput sequencing, microbial community, physicochemical factors

## Abstract

Overproduction of livestock manure can cause significant environmental challenges. Compost bedding (CB) is considered an effective approach for recycling the agricultural byproducts and improving the welfare of dairy cattle. During the CB preparing, the composition of microbial communities is usually altered; however, the patterns and drivers of CB microbial communities remains to be investigated. The current study aimed to explore the dynamics of bacterial and fungal communities during the various padded stages, using high throughput sequencing technology and qPCR. The relationships across physicochemical parameters, microbial community composition, and abundance were also evaluated. Sequencing results revealed that Proteobacteria, Bacteroidetes, Actinobacteria, and Firmicutes of bacteria, and Ascomycota of fungi as the major phyla found in CB. qPCR results showed a significant increase in the number of bacterial genome copies from 1.20 × 10^7^ to 3.35 × 10^7^ copies/gram of dry soil, while the number of fungal genome copies significantly increased from 8.43 × 10^4^ to 7.02 × 10^6^ copies/gram of dry soil. Linear discriminant analysis effect size (LEfSe) showed that Actinobacteria was the primary indicator in raw materials while the phylum Bacteroidetes was in the other padded stages. Dothideomycetes was significantly enriched in the initial stage of fungi, whereas Sordariomycetes, including a pathogen *Scedosporium prolificans*, was the major indicator in CB after 9 days of padding. Mantel test showed that pH significantly influenced bacterial community composition while temperature and total organic carbon (TOC) had a significant effect on fungal community structure. Redundancy analysis indicated that TOC, temperature, and water content had a significant effect on bacterial abundance while total nitrogen, water content, and pH significantly affected fungal abundance. Our finding contributed to the understanding of microbial community succession in CB across different padded stages, and suggests CB management by changing the bedding material every 7 days.

## Introduction

The large quantities of animal manure produced in livestock industry lead to serious environmental challenges. Compost bedding (CB) has been increasingly used worldwide to confine dairy cows (Weinberg et al., 2011; Favero et al., 2015; Eckelkamp et al., 2016). CB is typically prepared for improved cow comfort and relieves restrictions in standard stalls (Lobeck et al., 2011; Favero et al., 2015; Eckelkamp et al., 2016). Studies have shown that CB positively effects lactating cows, and may be beneficial for non-lactating cows as well (Endres and Barberg, 2007; Astiz et al., 2014; Biasato et al., 2019). Dried manure, straw, sawdust, and chopped woodchips constitute the most popular bedding material. Compost bedding is characterized by microbial decomposition of an organic substrate. Bed aeration promotes microbial activities that aid in the composition process, help maintain adequate moisture, and ensure a soft surface for cows (Black et al., 2014).

Since CB is mostly organic, one of the potential hazards for udder health is the concentration of environmental pathogens (Barberg et al., 2007a, b). Further, since the bedding is the immediate environment with which the cow is directly in contact, its lack of hygiene may lead to pathogenic conditions, such as mastitis, and toe and foot diseases in cows. Barberg and colleagues had reported a mean bedding concentration of total bacteria to be 9.1 × 10^6^ cfu/cc from a group of 12 CBs in Minnesota. Lobeck et al. (2012) had reported bedding concentrations of 1.4 × 10^3^, 280, and 3 × 10^6^ cfu/mL for coliforms, *Klebsiella* spp., and environmental streptococci, respectively (Lobeck et al., 2012). Other environmental pathogens that might be present in BC include *Nocardia*, *Pseudomonas*, *Prototheca*, and *Mycobacterium smegmatis* complex (Janosi et al., 2001; Condas et al., 2013; Ghielmetti et al., 2017). These pathogens affect the productivity and safety of cow milk consumed by humans. Traditional cultivation methods have been used to isolate and characterize the bacterial species. However, a large proportion of bacteria cannot be cultivated *in vitro*, which makes studying the natural microbial community in CB challenging (Mishra et al., 2014). The advent of high throughput sequencing has widened the possibility of detecting sequences of previously unknown microorganisms. Therefore, the dynamics of microbial communities in various composting systems has been studied, such as in cow manure and straw (Meng et al., 2019), poultry manure (Awasthi et al., 2018), and in vegetable waste (Liu et al., 2018). However, studies investigating the dynamics of microbial population in CB is lacking, especially for fungi. Thus, we used amplicon pyrosequencing of 16S rDNA and ITS (internal transcribed spacer) hypervariable regions to comprehensively define the bacterial and fungal composition, abundance, and structure of CB.

Compost bedding differs from conventional free stall and tie stall systems, since partitions are not present in the resting area. Previous studies had demonstrated that moisture, temperature, and organic components are the primary factors influencing the microbial structure in organic substrate fermentation, including pathogens (Chen and Chang, 2017; Xie et al., 2019); CB should, therefore, avoid the accumulation of moisture. A deeper understanding of bacterial and fungal population dynamics within the CB and its relationship with physicochemical characteristics is obviously required, to obtain a basis for better management of CB.

The objectives of this study were: (1) to investigate the bacterial and fungal communities, even pathogens, in different padded stages, and (2) to describe the relationship between microbial community and physicochemical characteristics of CB.

## Materials and Methods

### Study Sites and Sampling

The experiment was conducted in May 2018 in a commercial dairy farm in Linxia City, Gansu Province, China, housing 5,000 Holstein cows. The barn was a steel-frame structure, roofed with double slope-type color steel plate, metope is mixed brick to take window half open, and allowed communication with the peripheral playground. It adopted the double row head to spread the column type to raise, and had an automatic dung-clearing equipment.

Holstein cows, with similar health and body condition, were selected as test animals in the dairy farm, with management measures also being the same. The bedding material was composed of dried cow manure, fermented and sterilized with quicklime. The bedding mixture was aerated once or twice daily, which allow the incorporation of manure and urine into the compost, resulting in a dry laying surface for the cows.

Three barns were selected for this experiment. Samples were collected in the afternoon when the cows were out of the barn. In each barn, three bedding samples were collected from 2 to 10 cm below the surface of the pack to form a combined sample, which was subsequently placed in a sterile plastic tube and mixed thoroughly, and eventually analyzed for microbial population. The samples were kept at 4°C immediately after collection and stored at −20°C for long term storage. To analyze the physicochemical characteristics of CB, samples were taken from the same location, pooled together, mixed into one single sample, and kept in a plastic bag. The bedding samples were collected in the afternoon of Days 0, 1, 2, 5, 9, and 20.

### Measurement of Sample Characteristics

pH values were analyzed in 1:5 mixtures of sample: water using a pH meter (PT-10, Sartorius, Göttingen, Germany). Water content of the sample was determined gravimetrically, after drying 10 g of fresh soil in the oven at 105°C for 48 h. The total organic carbon (TOC) and total nitrogen (TN) contents were quantified with an automatic element analyzer (Elementar Vario-EL, Germany). Sample temperature was recorded three times at 5–10 cm depths using a geothermometer at each location.

### DNA Extraction, Amplification, and HiSeq Sequencing

Total DNA was isolated from the mixed samples, with three replicates, using the Soil DNA Isolation Kit (TIANGEN DP336, China), following the manufacturer’s instructions. We used the bacterial universal primers 338F and 806R to amplify the V3–V4 region of bacterial 16S rRNA genes. Meanwhile, the fungal universal primers 1737F and 2043R were used to amplify the ITS1 region. Amplification of the 16S rRNA genes and ITS1 region were conducted by an initial denaturation step at 94°C for 3 min, followed by 33 cycles of denaturation at 94°C for 30 s, annealing at 55°C for 30 s, and extension at 72°C for 30 s, with a final extension step at 72°C for 5 min. The amplicons were sequenced with Illumina HiSeq 2500 (Illumina, United States).

### Determination of Bacterial and Fungal Copy Numbers Using Quantitative Polymerase Chain Reaction

Real-time quantitative polymerase chain reaction (qPCR) was performed to estimate the abundance of bacteria and fungi in each sample. We used the universal bacterial 16S rRNA primers 515F (5**′**-GTGCCAGCMGCCGCGGTAA-3**′**) and 806R (5**′**-GGACTACHVGGGTWTCTAAT-3**′**) (Moreau and Rubin, 2017) and used a universal eukaryotic primer set from the hypervariable region of the V9 domain of the small subunit (SSU) in the ribosomal RNA (rRNA) gene complex-1380F (5**′**-CCCTGCCHTTTGTACACAC-3**′**) and 1510R (5**′**-CCTTCYGCAGGTTCACCTAC-3**′**) (Pagenkopp Lohan et al., 2016). All qPCR reactions, performed in triplicate, were conducted on a Stratagene Mx3000P (Agilent Technologies) following the manufacturer’s instructions. qPCR reactions involved using TB green SYBR super mix (Bio-Rad) and 1 μL of DNA extract. Standard curves were generated from the standard plasmid of ***Escherichia coli*** 16S rRNA and V9 domain of the SSU gene. To determine standard plasmid gene copy number per microgram of DNA, we measured the DNA concentration of each sample on a Qubit (Life Technologies, Grand Island, NY, United States) (Supplementary Table S1). The qPCR cycling parameters for bacteria were 30 s at 95°C, 40 cycles of 95°C for 5 s, 55°C for 30 s, and 72°C for 30 s, a dissociation stage of 95°C for 30 s and 55°C for 30 s and a final ramp-up to 95°C for 15 s. For fungi, cycling parameters were modified to 58°C annealing.

### Bioinformatics Analysis

The raw pyrosequencing reads were sorted with barcodes and quality trimmed using QIIME 1.8.0 (Caporaso et al., 2010). The parameters were as follows: paired-end reads with at least a 50-bp overlap and <5% mismatch were combined using FLASH (Magoc and Salzberg, 2011). A threshold of average quality scores > 30 over 5-bp window size was used to trim the unqualified sequences using BTRIM (Kong, 2011). Any joined sequence with ambiguous bases and lengths < 200 bp was discarded. All rarefaction curves approached a plateau, suggesting that the sequencing depth (30,000 sequences for bacteria and 160,000 sequences for fungi) for all samples was sufficient to cover microorganism community diversity (Supplementary Figure S1). Sequences were clustered into operational taxonomic units (OTUs) with a 97% identity threshold using UPARSE (Edgar, 2013), having the chimeras and all singletons being discarded. We determined taxonomic annotations using the Ribosomal Database Project Classifier with 80% confidence score (Wang et al., 2007). The 16S sequences were assigned using the Silva database (McDonald et al., 2012), while ITS sequences were assigned using the UNITE database (Abarenkov et al., 2010). To correct for sampling effects, we normalized all samples to an even number of sequences per sample before further analysis.

### Statistical Analyses

Alpha diversity metrics, including the Shannon–Wiener index and Simpson diversity index, were calculated using the “diversity” function in the Vegan package (version 2.4-4; Oksanen et al., 2013) of the software R version 3.3.2 (R Development Core Team, 2015). The bacterial and fungal community structures were visualized by non-metric multi-dimensional scaling (NMDS) ordinations based on the Bray–Curtis dissimilarity matrices using the Vegan package (Oksanen et al., 2013) of R software. One-way ANOVA was conduct to examine the changes of bacterial and fungal genome copy numbers over the various stages of CB. Pearson correlation coefficients were used to assess the associations between microbial alpha diversity and environmental factors, and the relationships across various environmental factors. A value of ***P*** < 0.05 was considered to be statistically significant. Estimation of alpha-diversity curve was performed across 18 samples. All statistical analyses were performed using SPSS 17.0.

Linear discriminant analysis (LDA) effect size (LEfSe) analysis was performed to reveal the significant ranking of abundant modules in different samples. A size-effect threshold of 2.0 in the logarithmic LDA score was used as discriminative functional marker. Relationships among the environmental variables and compositions of the bacterial and fungal community were examined with the redundancy analysis (RDA) function in the Vegan package of R software.

## Results

### Physicochemical Properties of Samples at Different Stages of Compost Bedding

Physicochemical properties of the manure samples are listed in Table 1. Total organic carbon ranged from 15.62 to 21.36 g/kg, TN ranged from 1.56 to 2.03 g/kg, and BA (Day 0) had the highest content of both TOC and TN. The C/N ratios ranged from 13.63 to 8.72. Temperature ranged from 31.5 to 20.9°C; BD (Day 5) had the highest temperature. Water content ranged from 2.72 to 1.92%; BF (Day 20) had the maximum moisture content. Soil pH ranged from 7.7 to 8.17. There was no significant correlation across the physicochemical properties (Supplementary Figure S2).

**TABLE 1:** The physicochemical factors of compost bedding samples at different padded stage.

Sample ID	Stage (d)	TOC %	TN %	C/N	Temperature (°C)	Water content (%)	pH
BA	0	21.36 ± 0.06	2.03 ± 0.03	10.52	25.76 ± 0.09	2.31 ± 0.17	7.70 ± 0.02
BB	1	21.26 ± 0.15	1.56 ± 0.00	13.63	25.17 ± 0.12	2.05 ± 0.37	8.17 ± 0.03
BC	2	18.78 ± 0.14	1.91 ± 0.06	9.83	25.87 ± 0.03	1.92 ± 0.16	8.13 ± 0.09
BD	5	15.62 ± 0.15	1.79 ± 0.00	8.72	31.50 ± 0.17	2.36 ± 0.05	7.89 ± 0.03
BE	9	16.50 ± 0.04	1.86 ± 0.00	8.87	22.10 ± 0.06	2.43 ± 0.14	8.14 ± 0.05
BF	20	18.84 ± 0.06	2.00 ± 0.03	9.42	20.90 ± 0.06	2.72 ± 0.01	8.09 ± 0.04

*Vaules represent means © standard error. TN, total nitrogen; TOC, total organic carbon; C/N, the ratio of TC to TN. Values in bold indicate significant correlations (*P* < 0.05).*

### Microbial Alpha Diversity Analysis

A total of 140,744 bacterial sequences and 939,861 fungal sequences were obtained from the 18 samples. When grouped at the level of 97% similarity, 2,952 OTUs were formed in bacteria. CB pad at Day 1 (BB) had the most OTUs (613), followed by CB pad at Day 2 (BC) (555), pad at Day 5 (BD) (533), pad at Day 9 (BE) (466), pad at Day 20 (BF) (399), and pad at Day 0 (BA) (386) (Table 2). A cubic regression was found (*r*^2^ = 0.547, *P* = 0.009, Supplementary Table S1). For fungi, 2,167 OTUs were formed. BC had the most OTUs (482), followed by BB (464), BE (387), BF (332), BD (317), and BA (185) (Table 3); a cubic regression was found (*r*^2^ = 0.596, *P* = 0.004, Supplementary Table S1). An OTU was defined as a read that shares 97% nucleotide-sequence identity. Alpha diversity, including Chao, Shannon, and Simpson indices, reflected the variation in microbial richness and diversity across the samples. The tendency of fungal richness and diversity increased first, followed by a decrease. Shannon and Simpson indices of bacteria were 4.27 and 0.033 respectively, in the unpadded CB (BA). The highest bacterial diversity was found in the CB padded 9 day (BE); Shannon and Simpson indices were 4.75 and 0.017 respectively. When the CB padding was 20 days old (BF), bacterial diversity decreased to its lowest levels, the Shannon and Simpson indices being 3.66 and 0.075, respectively.

**TABLE 2:** Pyrosequencing reads number, and alpha diversity of bacterial community at six stage of compost bedded pack.

Sample ID	Time (d)	Numbers of sequences	OTUs	Chao	Shannon	Simpson
BA	0	23532 ± 84	386 ± 59	361.86 ± 93.21	4.27 ± 0.27	0.033 ± 0.008
BB	1	22844 ± 77	613 ± 15	535.93 ± 45.4	4.59 ± 0.32	0.042 ± 0.025
BC	2	23893 ± 1164	555 ± 31	424.80 ± 18.6	3.82 ± 0.25	0.065 ± 0.016
BD	5	22924 ± 116	533 ± 60	540.19 ± 62.01	4.52 ± 0.36	0.032 ± 0.009
BE	9	23414 ± 132	466 ± 54	471.36 ± 55.21	4.75 ± 0.14	0.017 ± 0.002
BF	20	24137 ± 982	399 ± 45	372.96 ± 73.42	3.66 ± 0.19	0.075 ± 0.004

**TABLE 3:** Pyrosequencing reads number, and alpha diversity of fungal community at six stage of compost bedded pack.

Sample ID	Time (d)	Numbers of sequences	OTUs	Chao	Shannon	Simpson
BA	0	156609 ± 80	185 ± 26	79.43 ± 5.80	2.03 ± 0.42	0.221 ± 0.097
BB	1	154644 ± 1205	464 ± 91	255.96 ± 57.59	3.24 ± 0.24	0.084 ± 0.021
BC	2	155354 ± 4255	482 ± 70	215.73 ± 49.03	3.17 ± 0.35	0.093 ± 0.039
BD	5	156969 ± 1578	317 ± 25	129.70 ± 7.53	2.43 ± 0.15	0.145 ± 0.024
BE	9	157991 ± 1066	387 ± 1	194.69 ± 6.50	2.97 ± 0.02	0.123 ± 0.002
BF	20	158294 ± 976	332 ± 35	158.90 ± 13.25	2.42 ± 0.08	0.169 ± 0.021

For fungi, Shannon and Simpson indices were 2.03 and 0.221, respectively, in BA. The highest fungal diversity was found in BB, Shannon and Simpson indices being 3.24 and 0.084, respectively. With longer duration of CB padding, fungal richness and diversity were reduced. When the CB padding was 20 days old (BF), Shannon and Simpson indices were reduced to 2.42 and 0.169, respectively (Table 3).

Pearson correlation coefficient indicated TN to be negatively correlated with bacterial Chao 1 (*P* = 0.013) and fungal Shannon index (*P* = 0.032); and positively correlated with fungal Simpson index (*P* = 0.051). Water content was negatively correlated with fungal Shannon index (*P* = 0.028) (Supplementary Figure S3).

### Bacterial and Fungal Abundance and Composition

**FIGURE 1:**
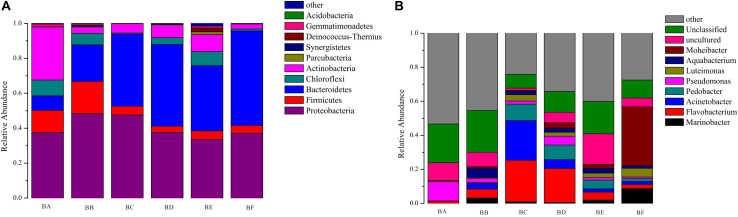
Community composition of bacteria at the tax level of Phylum **(A)** and Genus **(B)**.

A total of 19 phyla, 44 classes, 77 orders, 142 families, and 211 genera of bacteria were detected in the samples. According to the phylum assignment result, Proteobacteria was predominant bacterial in BA, BB, and BC (37.5–48.3%) followed by Actinobacteria in BA (30.3%). Bacteroidetes was the predominant bacterial phylum in BD to BF (37.3–54.1%), followed by Proteobacteria (33.5–37.6%) (Supplementary Table S2). At genus level, the most predominant were *Flavobacterium, Pseudomonas, and Acinetobacter* from BA to BE (Figure 1). It is noteworthy that, when the CB was padded for 20 days (BF), the bacterial community was obviously different, the dominant population being *Moheibacter* (34.65%), followed by *Marinobacter* (8.71%) (Figure 1).

**FIGURE 2:**
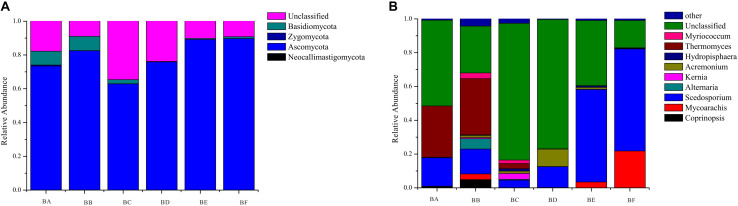
Community composition of fungi at the taxonomic level of Phylum **(A)** and Genus **(B)**.

A total of 4 phyla, 13 classes, 36 orders, 55 families, and 104 genera of fungi were detected in the samples. The most abundant fungal phylum was Ascomycota in all samples (62.9–89.8%) (Supplementary Table S3). Other phyla, including Zygomycota, Basidiomycota, and Neocallimastigomycota, accounted for only a minor fraction of fungal community composition (Figure 2). At the genus and species levels, fungal communities were dominated by Thermomyces in BA (30.33%) and BB (33.32%) and by Scedosporium in BE (75.82%) and BF (60.45%) (Figure 2).

**FIGURE 3:**
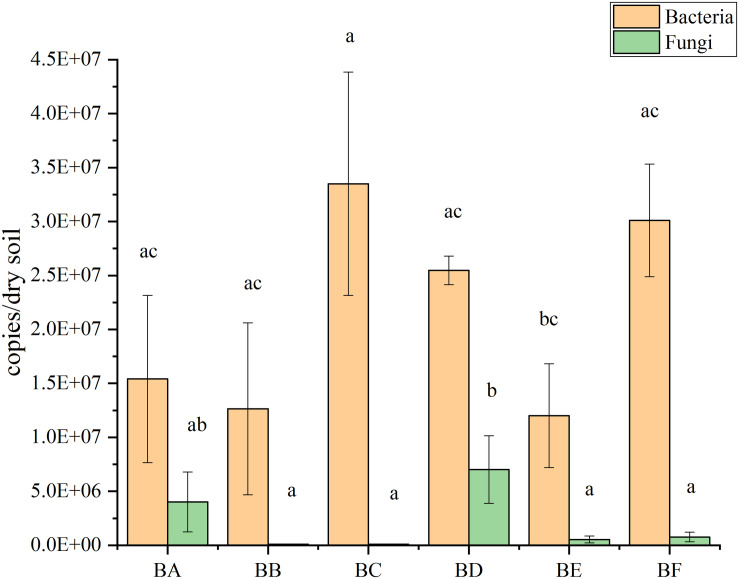
The abundance of bacteria and fungi at six stage of compost bedded pack.

qPCR results showed that bacterial abundance (number of 16S copies) was significantly increased from 1.20 × 10^7^ to 3.35 × 10^7^ copies/gram of dry soil. The abundance increased from BA to BC, where it reached maximum copies; then decreased from BD to BE, reaching at the minimum copies in BE, and finally increased again in BF (Figure 3). The abundance of the fungi (number of V9 copies) were significantly increased from 8.43 × 10^4^ to 7.02 × 10^6^ copies/gram of dry soil. The abundance increased to from BB to BD, reaching at the maximum copies in BD and then decreased from BE to BF (Figure 3).

### Bacterial and Fungal Communities

**FIGURE 4:**
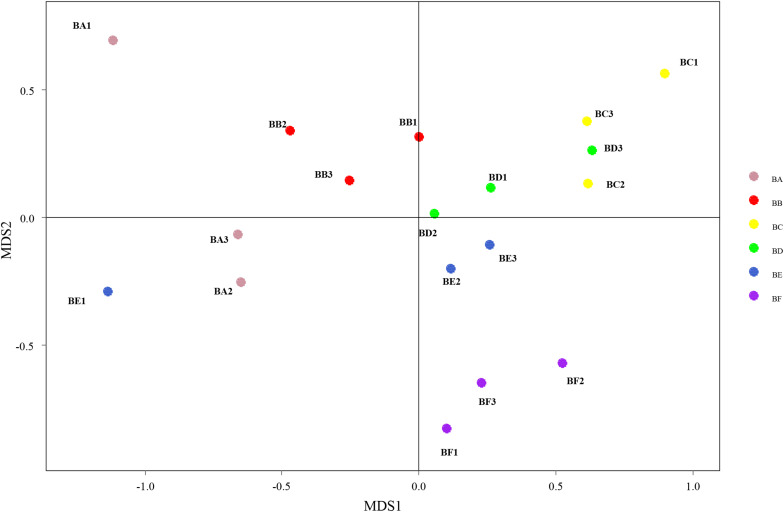
Non-metric multidimensional scaling (NMDS) biplot of Bray–Curtis dissimilarity matrix of bacteria at the genus level (*R*^2^ = 0.987).

**FIGURE 5:**
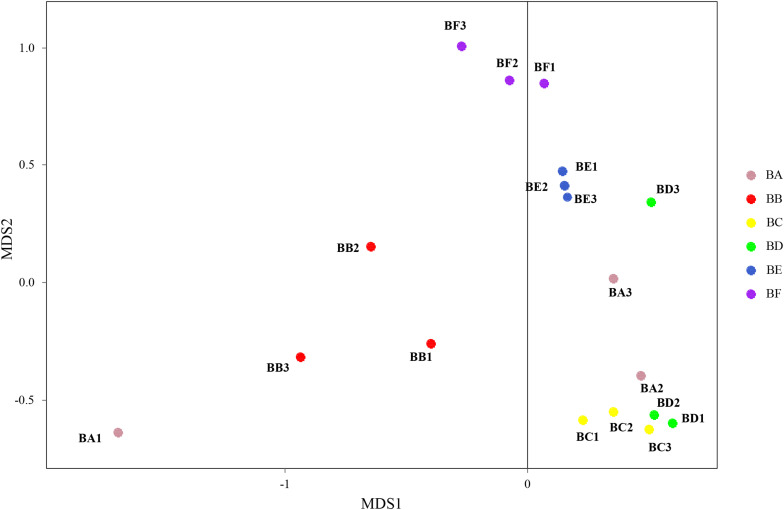
Non-metric multidimensional scaling biplot of Bray–Curtis dissimilarity matrix of fungus at the genus level (*R*^2^ = 0.99).

Differences in the composition of bacterial communities across all samples were identified with NMDS biplot (Figure 4). Notably, the CB pad at Day 2 and Day 5 were similar to that at Day 9 (BC, BD, and BE). The CB pad at Day 0 (BA), Day 1 (BB), and Day 20 (BF) represented independent community structure (Figure 4). Meanwhile, differences in the composition of fungal communities are shown in Figure 5. There were three community structures. The CB padding at Day 0 (BA) was similar to that at Day 2 (BC) and Day 5 (BD) while that at Day 9 (BE) and Day 20 (BF) were found. The CB padding at Day 1 (BB) represented independent community structure (Figure 5).

**FIGURE 6:**
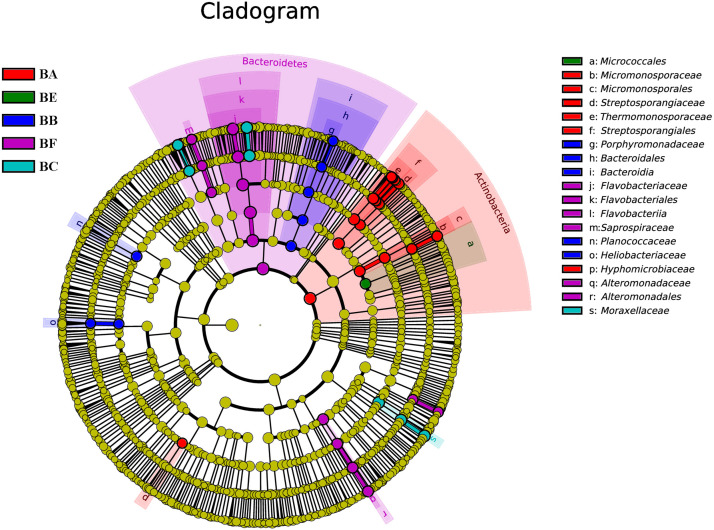
Bacterial taxa significantly differentiated between samples padded different stage identified by linear discriminant analysis effect size (LEfSe) using the default parameters.

To determine the functional community/communities in samples, LEfSe was conducted to identify the groups that display significant differences across the different bedding stages; the identified indicator groups are shown in a cladogram. The bacterial and fungal taxa varied during the entire process. For bacteria, Actinobacteria was significantly enriched in BA. There were 16 significantly abundant bacterial taxa including Streptosporangiaceae, Thermomonosporaceae, and Micromonosporaceae. Hyphomicrobiaceae was also significantly abundant in BA. Porphyromonadaceae belonged to the Bacteroidetes phylum, and Planococcaceae and Heliobacteriaceae were the most significantly abundant in BB. Flavobacteriaceae belonged to the Bacteroidetes phylum and Moraxellaceae was significantly abundant in BC. Meanwhile, only one taxon, Micrococcales, was overrepresented in BE. Bacteroidetes phylum was significantly enriched in BF. There were 14 significantly abundant bacterial taxa, including Flavobacteriaceae, *Moheibacter*, Alteromonadaceae, *Marinobacter*, and *Arenimonas*. Alteromonadaceae was also significantly abundant in BF (Figure 6).

**FIGURE 7:**
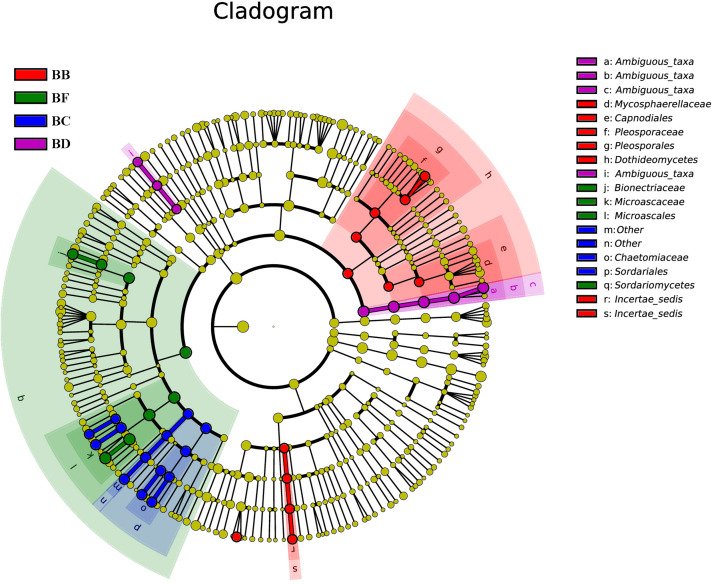
Fungal taxa significantly differentiated between samples padded different stage identified by linear discriminant analysis effect size (LEfSe) using the default parameters.

For fungi, there was no significantly different taxon across BA, BD, and BE. Dothideomycetes was overrepresented in BB. Sordariales belonged to Sordariomycetes, and was significantly abundant in BC. BF had Sordariomycetes, including *Scedosporium prolificans*, along with Microascaceae and Mycoarachis as the most abundant taxa (Figure 7).

### Association of Bacterial and Fungal Diversity and Abundance With Environmental Variables

**FIGURE 8:**
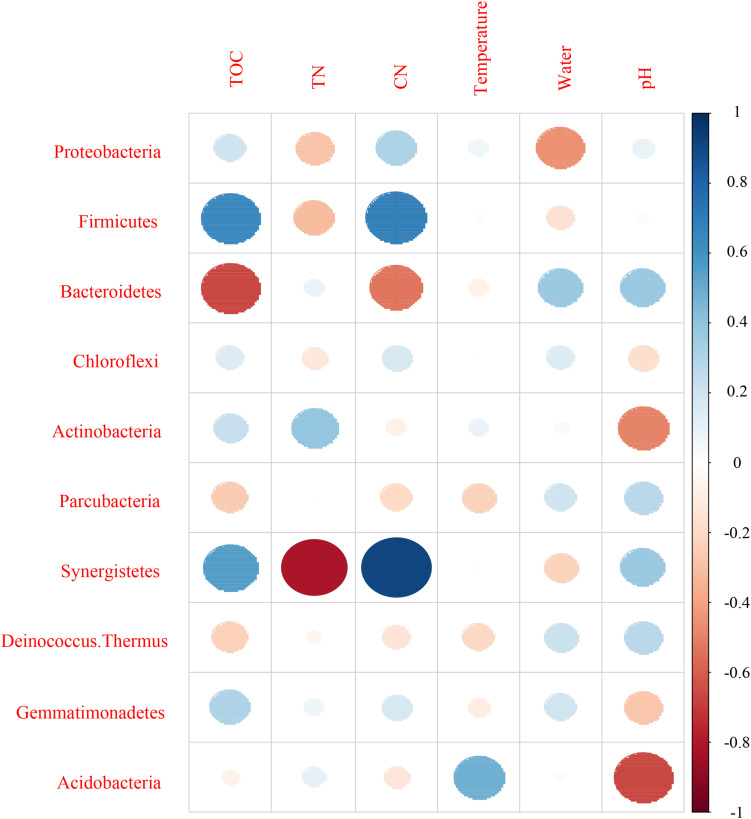
Pearson correlation between dominant bacterial phyla and environmental variables for all samples.

**FIGURE 9:**
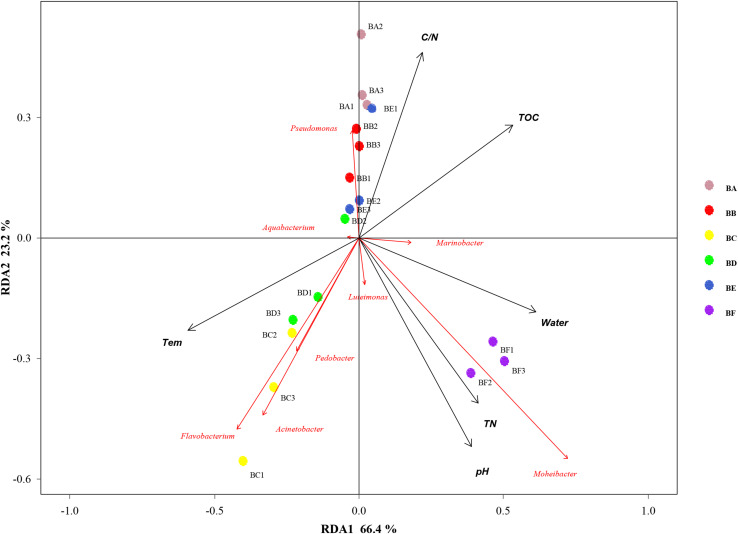
Bi-plot from the redundancy analysis (RDA) that shows the relationships between the bacterial community composition at the genus level and the environmental variables for all samples.

Mantel test indicated that pH could significantly influence bacterial community composition; TOC and temperature had a significant effect on fungal community structure (Table 4). Further, Pearson correlation was tested across the dominant bacterial and fungal phyla and environmental variables. For bacteria, the result showed TOC to be positively correlated with Firmicutes phylum (*P* < 0.01) and Synergistetes phylum (*P* < 0.05), whereas it was significantly negatively correlated with Bacteroidetes (*P* < 0.001). TN was negatively correlated with Synergistetes (*P* < 0.01). C/N was positively correlated with Firmicutes and Synergistetes (*P* < 0.01), and negatively correlated with Bacteroidetes (*P* < 0.05). Temperature was positively correlated with Acidobacteria (*P* < 0.05). Water content was negatively correlated with Proteobacteria (*P* < 0.05). pH was negatively correlated with Actinobacteria (*P* < 0.01) and Acidobacteria phyla (*P* < 0.05) (Figure 8 and Supplementary Table S4). Five environmental factors (i.e., temperature, TOC, TN, water content, and pH) were found to be correlated with the bacterial communities at genus level by RDA. Axis 1 explained 66.4% of the variance, and axis 2 explained 23.2% of the variance. TOC, temperature, and water content were the most important factors that significantly influenced the bacterial community at genus level. It is noteworthy that *Moheibacter* was the primary genus in BF, influenced by TN, pH, and water content (Figure 9).

**TABLE 4:** Relationships between bacterial and fungal community compositions at genus level and environmental variables CB revealed by Mantel test and partial Mantel test.

	Bacteria		Fungi	
Factors	Mantel test		Mantel test	
	*r*	*P*	*r*	*P*
TOC	0.056	0.249	0.175	**0.036**
TN	0.160	0.083	0.131	0.122
C/N	0.032	0.382	0.089	0.197
Temperature	–0.048	0.606	0.337	**0.011**
Water	0.091	0.218	–0.037	0.577
pH	0.260	**0.017**	0.131	0.117

*Values in bold indicate significant correlations (*P* < 0.05).*

**FIGURE 10:**
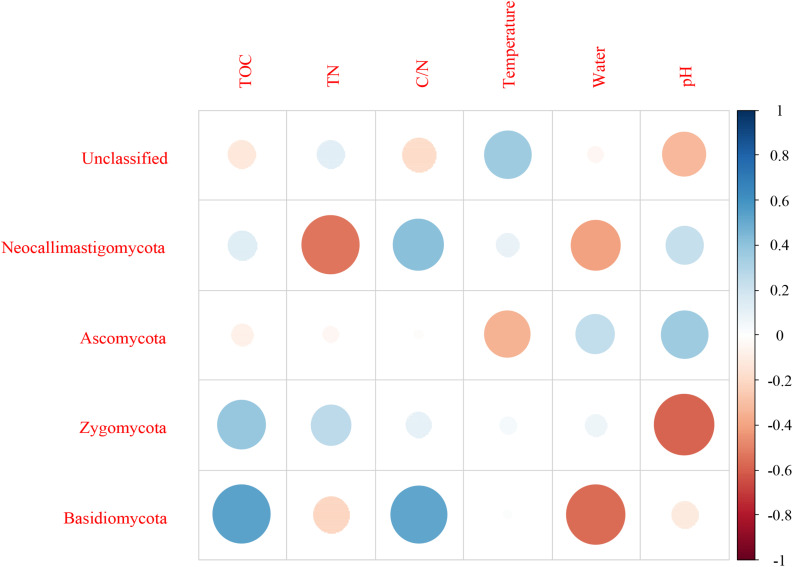
Pearson correlation between dominant fungal phyla and environmental variables for all samples.

**FIGURE 11:**
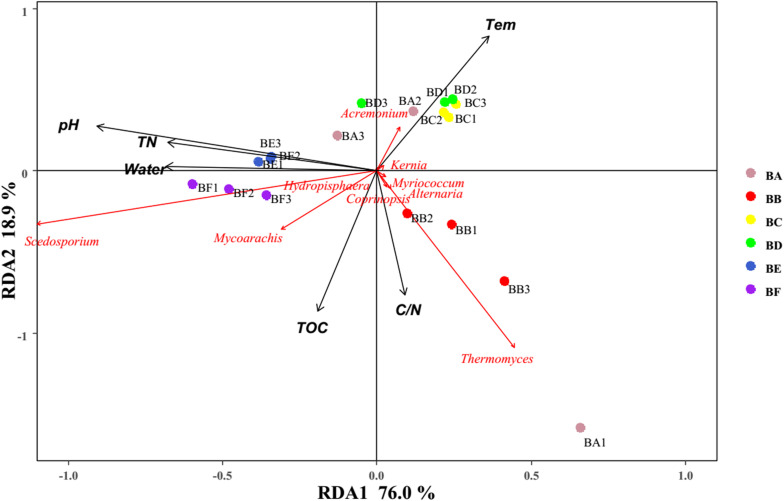
Bi-plot from the RDA that shows the relationships between the fungal community composition at the genus level and the environmental variables for all samples.

For fungi, correlation analysis results showed TOC to be positively correlated with Basidiomycota (*P* < 0.05). TN was negatively correlated with Neocallimastigomycota (*P* < 0.05). C/N was positively correlated with Basidiomycota (*P* < 0.05). Temperature was positively correlated with Acidobacteria (*P* < 0.05). Water content was negatively correlated with Basidiomycota (*P* < 0.01). pH was negatively correlated with Zygomycota (*P* < 0.05) (Figure 10 and Supplementary Table S5). As per the RDA result, axis 1 explained 76.0% of the variance, and axis 2 explained 18.9% of the variance. TN, water content, and pH significantly influenced the fungal community at genus level. *Thermomyces* was the primary genus in BB, influenced by TN, water content, and pH. *Scedosporium* was the primary genus in BE and BF, preferring high pH conditions (Figure 11).

Pearson correlation coefficient indicated that temperature was positively correlated with fungal abundance (*P* = 0.013). Environmental variables had no effect on bacterial abundance (Supplementary Figure S4).

## Discussion

Large-scale dairy cattle production yields a large amount of manure. Appropriate management is urgently required to reduce manure-related emission and environmental pollution. Compost bedding has been introduced to recycle the waste as a resource. However, research on the bacterial and fungal communities and diversity in dairy CB has been limited. The current study investigated CB microbiota and their relationships with the physicochemical factors driving microbial communities.

Our results have demonstrated that CB, at different stages, has different microbial communities, as illustrated by the microbial OTUs and alpha diversity indices (Table 2). The bacterial and fungal alpha diversity indices were found to increase and then decrease, reaching the lowest value in the bedding samples padded 20 days old. This increase might be attributed to the availability of easily useable organic substances that produce the fastest growing microorganism. Different microbial communities predominated different composting phases depending on the environment. In the entire CB padded progress, we found Proteobacteria and Bacteroidetes to be most dominant. Ren et al., 2016 had reported that in the co-composting of cow manure and rice straw, Proteobacteria and Bacteroidetes were the most abundant phyla. Lin et al. (2016) had reported that the highest protein activities in thermophilic swine manure digester were from Proteobacteria. These results indicated that Proteobacteria and Bacteroidetes played a vital role in degradation of organic materials. Bacteroidetes promotes digestion and increases utilization of complex carbohydrates (Partanen et al., 2010), such as *Flavobacterium* (phylum Bacteroidetes) which influences denitrification during composting (Bhatia et al., 2013). *Pseudomonas* (phylum Proteobacteria) could degrade proteins, starch, and other organisms (Chen et al., 2017). Firmicutes were obtained from gut microbiota, and therefore were relatively abundant in BA and BB (Chen et al., 2017). We also found that Actinobacteria was the secondary phylum and major indicator of the unused bedding stage (Figures 1, 6). Actinobacteria is a Gram-positive group of bacteria, often the major bacterial group at the thermophilic stage of composting. At high concentrations, Actinobacteria start to decompose hemi-cellulose, cellulose, and lignin (Steger et al., 2007); increase of the phylum may have some correlation with the degradation of organic matter (Ventura et al., 2007). Moheibacter accounted for the maximum component in BF (Figure 1). Moheibacter is a member of the Flavobacteriaceae family, and often found in a biogas plant (Schauss et al., 2016).

For fungi, a high concentration of Ascomycota often indicates severe decomposition. Ascomycota have been reported in composting of cow manure, food and garden waste, and sewage sludge (Langarica-Fuentes et al., 2014; Wang et al., 2018; Meng et al., 2019). Ascomycota can secrete many kinds of cellulose- and hemicellulose-degrading enzymes, which can efficiently utilize nutrient elements in compost. Thermomyces lanuginosus was the most dominant at the initial stage (CB padded Day 0 and Day 1) (Figure 7). It can produce thermostable hemicellulases, and is likely to function in hemicellulose degradation during the composting process (Gu et al., 2017). Sordariomycetes was the indicator group in BF, and increased continuously from beginning to completion (Figure 7), in the process of co-composting of organic wastes. Sordariomycetes can tolerate higher temperatures and lower moisture conditions due to the specific microbial structure and the spore-like cellular features (Duan et al., 2019). *Scedosporium prolificans* (*Lomentospora prolificans*) became the major species when CB padded Day 9. The large proportion of *S. prolificans* in CB, may cause public health hazard since it may cause serious illness. Therefore, we recommend that the CB to be refreshed periodically every 7 days.

From the qPCR result (Figure 3), we found that number of bacterial genomic copies was more than those of fungi by 1.7–397 times. The excess of bacterial genome copies as compared with fungal copies has been previously observed (Galitskaya et al., 2017). In the CB padded stage, bacterial numbers grew significantly as the degradable organic compounds increased. Temperature was positively correlated with fungal abundance (Supplementary Figure S4). This finding was consistent with previous studies showing a decrease in the amount of fungi during thermophilic composting stages and a subsequent increase during cooling (Langarica-Fuentes et al., 2014).

With the changes in physicochemical parameters and degradation of bedding materials, padding process significantly affects microbial population. NMDS results revealed that microbial community compositions were significantly different across the different padded stages. Interestingly, we found that distance matrices of fungal community compositions were closer than bacteria, indicating that a high similarity of fungal community compositions was shared across the different composting stages. This result was also validated by alpha diversity index analysis, since bacterial Shannon diversity index was higher than that of fungi in this study. In addition, bacterial community compositions in the CB padded for 20 days was significantly different from others. This correlated with the nature and nutritional status of the CB system during the padding phases, thus suggesting bacterial species to be role-specific and environment-sensitive. This result agrees with the previous studies by Ren et al. (2016) and Meng et al. (2019) who had reported similarity of bacterial diversity at both cooling and maturation stages during cow manure and corn/rice straw composting. CB, padded for 9 and 20 days, indicated the sharing of a high similarity of fungal community compositions. It implies that biological progress is significantly different from the latter stage.

The alteration of physicochemical parameters has both direct and indirect influence on the activities of microorganisms. In this study, Mantel test, Pearson correlation, and RDA analysis revealed the abundance and community composition of bacteria and fungi to be significantly affected by different physicochemical parameters of CB. A significant relationship of TOC and temperature was found with fungal abundance, but not with bacterial abundance, suggesting that growth of fungi was more sensitive to changes in TOC and temperature than that of bacteria. However, pH significantly influenced the bacterial community composition rather than fungal community, which indicates that succession of bacterial community was more sensitive to pH variation than that of fungal community. RDA analysis showed TOC, temperature, and water content to be the most vital factors that significantly influence the bacterial community, and all were found to significantly influence the fungal community as well. Rousk et al. (2010) had reported that pH would have a weaker influence on fungi than on bacteria, since individual species of fungi have a broader range of pH for optimal growth compared to individual species of bacteria. Previous studies also investigated pH as an important factor in determining bacterial community diversity (Brockett et al., 2012; Zhalnina et al., 2015). In Pearson correlation and RDA analysis, pH was revealed as a major factor driving the changes of microorganism, and correlated with Actinobacteria, Acidobacteria, and Zygomycota phyla (all *P* < 0.05). TOC was found to significantly correlate with the distribution of bacterial and fungal communities. This result was consistent with the report by Hu et al. (2017) and Meng et al. (2019). TOC is an essential nutrient for microbial growth and has been reported as an important factor affecting microbial community structure and metabolic type (Hu et al., 2017). In support of this deduction, further analysis showed significant correlation among TOC and the relative abundance of different phyla such as Firmicutes, Synergistetes, Bacteroidetes, and Basidiomycota (all *P* < 0.05). Interestingly, TN significantly influenced the abundance and community composition of bacteria and fungi, which indicated that both bacteria and fungi played key roles in the transformation of nitrogen in composting. Therefore, rates of nutrient transformation and compost maturation are processes mainly sponsored by activities of microorganisms (Meng et al., 2019). Significant correlations of TN with the relative abundance of different phyla such as Synergistetes, Neocallimastigomycota (all *P* < 0.05), and microbial alpha diversity were seen. With higher substrate C/N benefiting fungal growth (Eiland et al., 2001), abundance of *Scedosporium* increased during the CB padding process. In this study, temperature and water content affected the microbial communities, in accordance with the response to environmental variables during cow manure composting (Hu et al., 2017; Meng et al., 2019). Temperature determines the rate of organic matter decomposition. Water content affects the amount of dissolved organic matter and enzyme activities directly, thereby influencing the community composition of bacteria and fungi (Hu et al., 2017). Significant correlation was seen between water content and relative abundance of different phyla, such as Proteobacteria and Basidiomycota (all *P* < 0.05), and microbial alpha diversity.

In summary, this study provided insights into the distribution patterns and drivers of microbial communities of CB. Our results revealed that bacterial and fungal alpha diversity increased first and reduced subsequently. Bacterial community compositions in the 20-day padded CB were significantly different from others while fungal community in 9- and 20-day padded CB shared a high similarity in composition. Our results also demonstrated that Proteobacteria, Bacteroidetes, and Firmicutes were dominant phyla of bacteria and Ascomycota was the dominant phylum of fungi. The qPCR results showed that the number of bacterial genome copies significantly increased from 1.20 × 10^7^ to 3.35 × 10^7^ copies/gram of dry soil and the number of fungal genome copies also increased from 8.43 × 10^4^ to 7.02 × 10^6^ copies/gram per dry soil. Actinobacteria were the indicators in the raw material and Bacteroidetes in the other padded stage of bacteria. Dothideomycetes was significantly enriched in the initial stage of fungi, whereas Sordariomycetes, including a pathogen *S. prolificans*, was the major indicator in CB after 9 days of padding. Our results showed that TOC, temperature, water content, and pH had a significant effect on bacterial abundance while TN, along with temperature, TOC, water content, and pH significantly affected fungal abundance.

## Data Availability Statement

The bacterial and fungal DNA sequences in our manuscript have been deposited in the SRA of the NCBI database under the SRA accession PRJNA602596, Biosample accession SAMN13899480–13899497.

## Author Contributions

LS did contributions to acquisition and analysis of data, drafted and revised the manuscript. SG did contributions to conception and design for the study. XH and JL collected and recruited materials. QX and XG did experiment and coordinated to acquire the data. ZZ coordinated to acquire the data and revise the manuscript. YL modified the pictures throughout the manuscript. All authors reviewed and approved the final manuscript.

## Conflict of Interest

The authors declare that the research was conducted in the absence of any commercial or financial relationships that could be construed as a potential conflict of interest.
