# Corrigendum: Microbial Community and Its Association With Physicochemical Factors During Compost Bedding for Dairy Cows

**DOI:** 10.3389/fmicb.2020.590323

**Published:** 2020-10-23

**Authors:** Likun Sun, Xiangmin Han, Jianshu Li, Zhidong Zhao, Yuzhen Liu, Qiming Xi, Xinyu Guo, Shuangbao Gun

**Affiliations:** ^1^College of Animal Science, Gansu Agricultural University, Lanzhou, China; ^2^Gansu Provincial Engineering Research Center for Animal Waste Utilization, Gansu Agricultural University, Lanzhou, China; ^3^Grassland Science College of Gansu Agricultural University, Lanzhou, China

**Keywords:** cow manure, compost bedding, high throughput sequencing, microbial community, physicochemical factors

There is an error in the Funding statement. The corrected acknowledgment of the Funder should read “The scientific research start-up funds for openly recruited doctors (2017RCZX-19).”

The authors apologize for this error and state that this does not change the scientific conclusions of the article in any way. The original article has been updated.

