# Supplementary material for: Microbial Community and Its Association With Physicochemical Factors During Compost Bedding for Dairy Cows
Source: Front Microbiol. 2020 Feb 21;11:254. doi: 10.3389/fmicb.2020.00254 (PMC7047772; doi:10.3389/fmicb.2020.00254)
Supplement: Supplementary file 1 [file Data_Sheet_1.docx]

Figure S1. Rarefaction curves based on 16S rRNA and ITS sequences among the different samples.

Table S1 The DNA concentration of each sample measuring by Qubit.

| Sample ID | Concentration (ng/μL) | Sample ID | Concentration (ng/μL) | Sample ID | Concentration (ng/μL) |
| --- | --- | --- | --- | --- | --- |
| BA1 | 37.5 | BA2 | 56.1 | BA3 | 14.4 |
| BB1 | 21.8 | BB2 | 18.5 | BB3 | 26.0 |
| BC1 | 33.2 | BC2 | 30.9 | BC3 | 37.0 |
| BD1 | 47.0 | BD2 | 68.4 | BD3 | 78.6 |
| BE1 | 69.4 | BE2 | 105.9 | BE3 | 16.7 |
| BF1 | 48.5 | BF2 | 117.7 | BF3 | 88.8 |

Table S2 The curve estimation of alpha-diversity.

| Alpha-diversity | Linear |  | Quadratic |  | Cubic |  |
| --- | --- | --- | --- | --- | --- | --- |
|  | r^2^ | *P* | r^2^ | *P* | r^2^ | *P* |
| b- OTUs | 0.033 | 0.470 | 0.433 | 0.014 | 0.547 | **0.009** |
| b- Chao 1 | 0.000 | 0.969 | 0.220 | 0.155 | 0.220 | 0.308 |
| b- Shannon | 0.027 | 0.511 | 0.087 | 0.507 | 0.233 | 0.280 |
| b- Simpson | 0.035 | 0.457 | 0.057 | 0.644 | 0.381 | **0.074** |
| f- OTUs | 0.021 | 0.569 | 0.339 | 0.045 | 0.596 | **0.004** |
| f- Chao 1 | 0.007 | 0.737 | 0.160 | 0.271 | 0.397 | **0.062** |
| f- Shannon | 0.001 | 0.901 | 0.245 | 0.122 | 0.374 | **0.079** |
| f- Simpson | 0.003 | 0.818 | 0.199 | 0.190 | 0.280 | 0.191 |


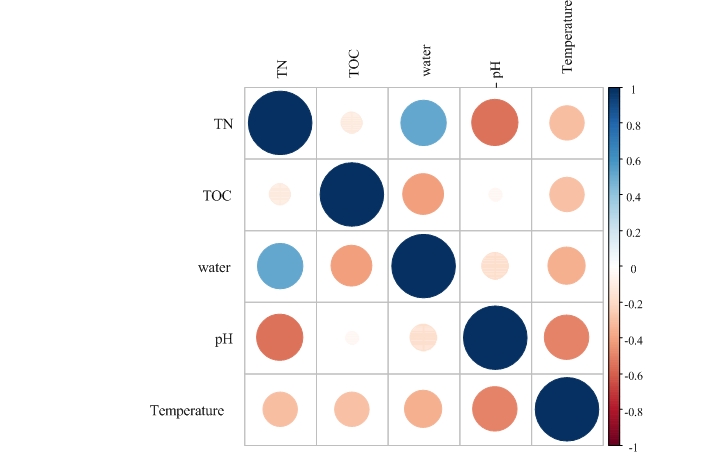


Figure S2. Pearson correlation between different environmental variables.

| Sample ID | Time (d) | Proteobacteria | Firmicutes | Bacteroidetes | Chloroflexi | Actinobacteria | Parcubacteria | Synergistetes | Deinococcus-Thermus | Gemmatimonadetes | Acidobacteria | other |
| --- | --- | --- | --- | --- | --- | --- | --- | --- | --- | --- | --- | --- |
| BA | 0 | 37.5±17.1 | 12.6±5.9 | 8.2±4.3 | 9.1±4.2 | 30.3±19.0 | 0.0±0.0 | 0.0±0.0 | 0.1±0.0 | 1.6±0.5 | 0.2±0.0 | 0.2±0.0 |
| BB | 1 | 48.3±11.6 | 18.4±3.0 | 20.9±7.0 | 6.6±1.7 | 3.5±0.7 | 0.0±0.0 | 0.5±0.0 | 0.4±0.0 | 0.7±0.2 | 0.0±0.0 | 0.3±0.0 |
| BC | 2 | 47.5±1.8 | 4.9±0.4 | 41.3±2.1 | 0.6±0.1 | 5.3±1.3 | 0.0±0.0 | 0.0±0.0 | 0.0±0.0 | 0.0±0.0 | 0.0±0.0 | 0.0±0.0 |
| BD | 5 | 37.6±2.1 | 3.5±0.9 | 46.7±5.4 | 3.8±2.4 | 7.3±1.9 | 0.0±0.0 | 0.0±0.0 | 0.2±0.1 | 0.2±0.1 | 0.2±0.0 | 0.2±0.0 |
| BE | 11 | 33.5±6.9 | 4.8±3.2 | 37.3±1.7 | 8.0±6.0 | 9.7±2.0 | 1.6±1.6 | 0.0±0.0 | 2.1±2.0 | 1.1±0.8 | 0.0±0.0 | 1.5±0.6 |
| BF | 20 | 37.1±3.5 | 4.4±2.5 | 54.1±2.7 | 1.1±0.7 | 2.8±1.2 | 0.0±0.0 | 0.0±0.0 | 0.2±0.0 | 0.0±0.0 | 0.0±0.0 | 0.4±0.2 |

Table S3 The relative abundances (%) of top ten bacterial phyla in bedding of different padded time. Values at individual sites are mean values of three replicated samples (mean ± standard error).

Table S4 The relative abundances (%) of fungal phyla in bedding of different padded time. Values at individual sites are mean values of three replicated samples (mean ± standard error).

| Sample ID | Time (d) | Ascomycota | Basidiomycota | Zygomycota | Neocallimastigomycota | Unclassified |
| --- | --- | --- | --- | --- | --- | --- |
| BA | 0 | 73.5±12.4 | 8.2±6.5 | 0.6±0.3 | 0.0±0.0 | 17.7±9.7 |
| BB | 1 | 82.5±4.4 | 8.5±4.5 | 0.0±0.0 | 0.0±0.0 | 8.9±1.4 |
| BC | 2 | 62.9±16.7 | 2.5±0.6 | 0.0±0.0 | 0.0±0.0 | 34.5±16.8 |
| BD | 5 | 75.9±7.0 | 0.2±0.0 | 0.0±0.0 | 0.0±0.0 | 23.8±7.0 |
| BE | 11 | 89.2±2.0 | 0.5±0.0 | 0.0±0.0 | 0.0±0.0 | 10.1±2.0 |
| BF | 20 | 89.8±1.9 | 1.0±0.1 | 0.0±0.0 | 0.0±0.0 | 9.2±1.8 |

Table S5 Pearson correlation coefficient (r) between dominant bacterial phyla and environmental variables for all samples.*indicate significant correlation (*** indicates *P*<0.001; **indicates *P*<0.01;*indicates *P*<0.05).

|  | Proteobacteria | Firmicutes | Bacteroidetes | Chloroflexi | Actinobacteria | Parcubacteria | Synergistetes | Deinococcus-Thermus | Gemmatimonadetes | Acidobacteria | other |
| --- | --- | --- | --- | --- | --- | --- | --- | --- | --- | --- | --- |
| TOC | 0.203 | 0.650** | -0.661** | 0.147 | 0.221 | -0.255 | 0.554* | -0.238 | 0.308 | -0.061 | -0.212 |
| TN | -0.283 | -0.316 | 0.082 | -0.121 | 0.394 | 0.006 | -0.817** | -0.052 | 0.079 | 0.108 | -0.062 |
| C/N | 0.313 | 0.687** | -0.534* | 0.172 | -0.076 | 0.192 | 0.919** | -0.143 | 0.171 | -0.137 | -0.118 |
| Temperature | 0.070 | -0.020 | -0.076 | 0.000 | 0.081 | -0.223 | -0.002 | -0.205 | -0.095 | 0.486* | 0.183 |
| water | -0.541** | -0.092 | 0.327 | 0.081 | 0.112 | 0.163 | -0.266 | 0.166 | 0.193 | -0.008 | 0.152 |
| pH | -0.029 | 0.375 | 0.327 | -0.177 | -0.492* | -0.336 | 0.374 | 0.279 | -0.274 | -0.666** | 0.263 |

Table S6 Pearson correlation coefficient (r) between dominant fungal phyla and environmental variables for all samples.*indicate significant correlation (*** indicates *P*<0.001; **indicates *P*<0.01;*indicates *P*<0.05).

|  | Neocallimastigomycota | Ascomycota | Zygomycota | Basidiomycota | other |
| --- | --- | --- | --- | --- | --- |
| TOC | 0.134 | -0.078 | 0.382 | 0.537* | -0.129 |
| TN | -0.536* | -0.043 | 0.266 | -0.211 | 0.121 |
| C/N | 0.417 | -0.140 | 0.107 | 0.521* | -0.185 |
| Temperature | 0.091 | -0.346 | 0.047 | 0.012 | 0.358 |
| water | -0.416 | 0.282 | 0.063 | -0.601** | -0.069 |
| pH | 0.239 | 0.359 | -0.581* | -0.118 | -0.321 |


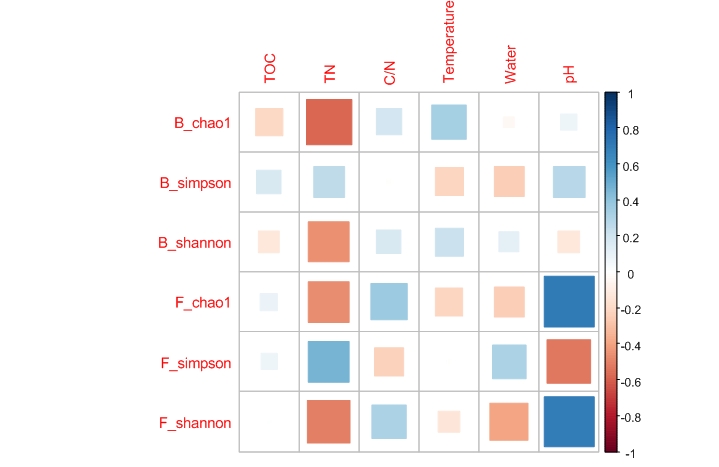


Figure S3. Pearson correlation between bacterial and fungal alpha diversity and environmental variables for all samples.


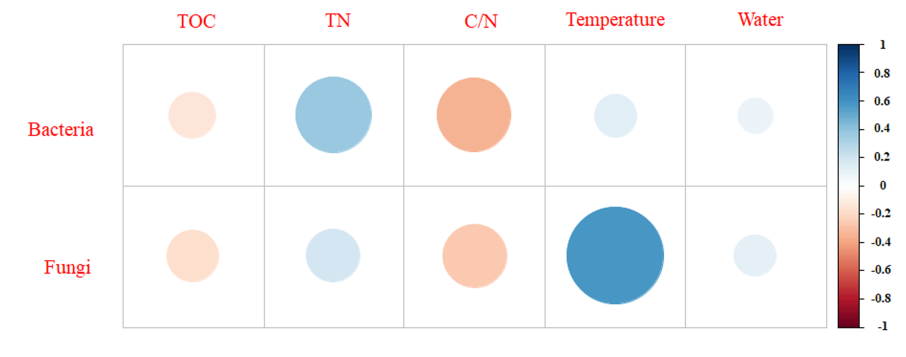


Figure S4. Pearson correlation between bacterial and fungal abundance and environmental variables for all samples.
